# Role of Quercetin in Diabetic Cardiomyopathy

**DOI:** 10.3390/plants14010025

**Published:** 2024-12-25

**Authors:** Nor Hidayah Mustafa, Hawa Nordin Siti, Yusof Kamisah

**Affiliations:** 1 Centre for Drug and Herbal Development, Faculty of Pharmacy, Universiti Kebangsaan Malaysia, Kuala Lumpur 50300, Malaysia; norhidayahmustafa23@gmail.com; 2 Department of Basic Medical Sciences, Faculty of Medicine, Universiti Sultan Zainal Abidin, Kuala Terengganu 20400, Malaysia; hawanordin@gmail.com; 3 Department of Pharmacology, Faculty of Medicine, Universiti Kebangsaan Malaysia, Kuala Lumpur 56000, Malaysia; 4 Cardiovascular and Pulmonary Research Group, Universiti Kebangsaan Malaysia, Bangi 43600, Malaysia

**Keywords:** quercetin, diabetes, heart disease, oxidative stress, inflammation, calcium regulation, apoptosis, fibrosis, heart function

## Abstract

Diabetic cardiomyopathy is a significant and severe complication of diabetes that affects a large portion of the global population, with its prevalence continuing to rise. Secondary metabolites, including quercetin, have shown promising effects in mitigating the progression of diabetic cardiomyopathy by targeting multiple pathological mechanisms, including impaired insulin signaling, glucotoxicity, lipotoxicity, oxidative stress, inflammation, fibrosis, apoptosis, autophagy, mitochondrial dysfunction, cardiac stiffness, and disrupted calcium handling. Addressing these mechanisms is crucial to prevent left ventricular diastolic and systolic dysfunction in advanced stages of diabetic heart disease. Scientific evidence has highlighted the cardioprotective properties of quercetin at both the myocardial and cellular/molecular levels in diabetic models. Therefore, this review aims to present a comprehensive overview of the proposed mechanisms underlying quercetin’s beneficial effects, providing valuable insights that could inform future drug discovery efforts specific to diabetic cardiomyopathy.

## 1. Introduction

Cardiovascular–kidney–metabolic (CKM) syndrome, a subset of metabolic syndrome, arises from disruptions in normal body physiology and has become a major global health concern. It involves the interconnected dysfunction of the cardiovascular system, kidneys, and metabolic pathways, often linked to conditions like diabesity (co-existing diabetes and obesity). Diabetes mellitus, characterized by chronic hyperglycemia, contributes to complications affecting various organs, including cardiovascular disease, retinopathy, and kidney dysfunction, impacting approximately 422 million individuals worldwide [1].

Diabetes mellitus is a disorder that occurs due to the defective pancreatic secretion of insulin or insulin resistance. Two major types of diabetes mellitus are type 1 diabetes mellitus, which is caused by pancreatic beta cell destruction, and type 2 diabetes mellitus, which primarily occurs due to insulin resistance whereby our body does not effectively respond to insulin [2]. It is well recognized for increasing the risk of heart failure, a condition referred to as diabetic cardiomyopathy. This cardiomyopathy is characterized by changes in both cardiac structure, such as enlargement, and function, including impaired contractility [3]. It is believed that continued exposure of the heart to hyperglycemia augments O-linked β-N-acetylglucosaminylation (O-GlcNAcylation) and the production of the maladaptive non-enzymatic advanced glycation end-products (AGEs) of cardiomyocyte proteins, thereby accelerating oxidative stress. This maladaptive process undermines cardiac function, contributing to stiffness and impairments in contractility and relaxation, which in turn contribute to the progression of diabetic cardiomyopathy [4,5].

**Figure 1:**
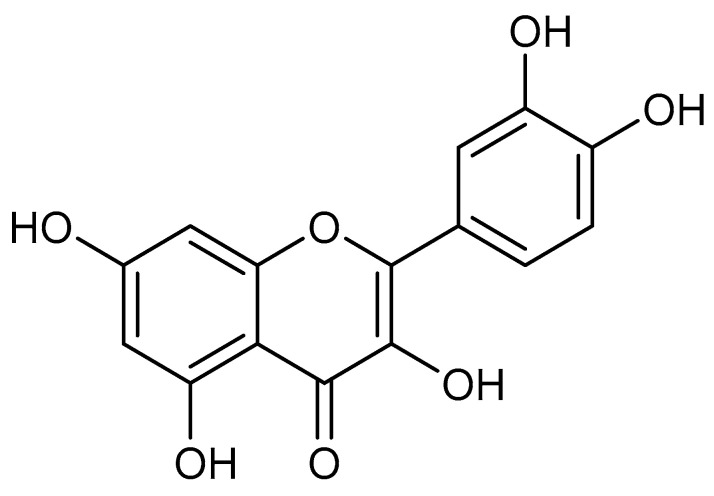
Molecular structure of quercetin.

Plants have long served as a source of medicinal products for various ailments [6]. The effects of many plant extracts and secondary metabolites, such as quercetin, have been investigated in relation to diabetes [7,8,9] and diabetic cardiomyopathy [10,11]. Quercetin (Figure 1) is a flavonoid found in many plants, including apples [12], berries [13], tea [14], and stink beans [15]. It exerts various pharmacological effects, such as antioxidant [16], anti-inflammatory [17], anti-apoptotic [18], and antifibrotic [10] activities, which may contribute to its beneficial effects on diabetic cardiomyopathy.

**Figure 2:**
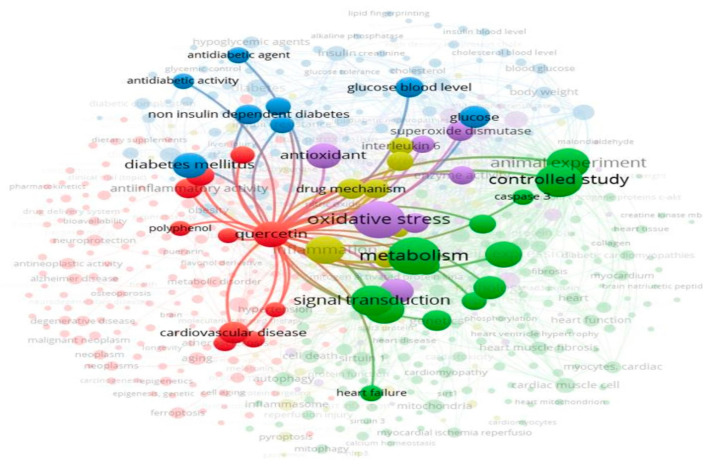
Keyword network analysis in the mechanism of diabetic cardiomyopathy. The circle size indicates keyword frequency, while the connecting links represent relevance and relationships.

Quercetin has garnered significant attention in recent years. By the end of 2024, over 29,000 articles on quercetin were indexed in the Web of Science, Scopus, and PubMed, with more than 1,000 focusing on its effects on cardiovascular health, based on data retrieved from searches conducted up to November 2024. Figure 2 presents network visualizations of the publication trends and keyword associations related to quercetin in the context of diabetic cardiomyopathy. Despite this growing body of work, the molecular and mechanobiological aspects of quercetin in diabetic cardiomyopathy remain unclear. Understanding these mechanisms is crucial for guiding future research. This review aims to summarize and critically evaluate quercetin’s pharmacological mechanisms in this context.

## 2. Pharmacokinetics of Quercetin: Insights into Bioavailability and Metabolism

The absorption of quercetin after oral administration is highly variable, ranging from 0% to over 50% of the administered dose [19]. The main reasons for the poor bioavailability of quercetin are its metabolism in the gut and the induction of efflux transporter P-glycoprotein, which reduces its intestinal absorption. The type of sugar moiety attached to quercetin influences the absorption of quercetin glycoside in the small intestine. Quercetin aglycone is lipophilic and primarily absorbed by intestinal cells through passive diffusion, whereas other quercetin derivatives may have higher polarity and require transporters. Sodium-dependent glucose co-transporter-1 (SGLT-1), expressed on the intestinal epithelial cells, is one of the transporters involved in the absorption of quercetin and its glycosides [20].

The metabolism of quercetin has been extensively reviewed by Muñoz-Reyes et al. [21]. In brief, the hydrolysis of quercetin glycosides primarily occurs in intestinal cells, facilitated by lactase-phlorizin hydrolase at the cell surface and intracellular β-glucosidases. This deconjugation process produces quercetin aglycone, increasing its lipophilicity. In enterocytes, quercetin undergoes enzymatic glucuronidation, methylation, and sulfation, resulting in a variety of metabolites. The main metabolites that enter the portal vein in humans are quercetin 3-O-glucuronide and quercetin 3′-O-sulfate. Further glucuronidation, methylation, and sulfation processes occur in the liver, generating metabolites such as quercetin diglucuronide, quercetin sulfate monoglucuronide, and isorhamnetin 3-O-glucuronide, which then enter the systemic circulation. In the bloodstream, over 90% of quercetin metabolites are extensively bound to plasma proteins, predominantly albumin [22]. As a result, only a small fraction of free quercetin metabolites are available for tissue distribution, which varies across species [23].

## 3. Effects of Quercetin on Hyperglycemia and Dyslipidemia

Hyperglycemia develops when the release of insulin from the pancreas, which converts glucose to glycogen, is impaired, most of the time due to the damage and loss of pancreatic islet β cells [24] as well insulin resistance [25]. Key enzymes, such as α-glucosidase, α-amylase, and dipeptidyl peptidase-4 enzymes, can control postprandial hyperglycemia and reduce the risk of developing diabetes [26]. Quercetin exhibits α-amylase- [27,28], α-glucosidase- [25,27], and dipeptidyl peptidase-4-inhibiting properties [29,30,31], which are the therapeutic targets for type 2 diabetes [26,32].

Numerous studies have investigated the effects of quercetin on blood glucose levels, yielding inconsistent results. Some studies reported no change in blood glucose or insulin levels with quercetin treatment, while others demonstrated positive effects (Table 1). The reasons for the negative findings remain unclear. The positive effects of quercetin on hyperglycemia are likely due to its antioxidant properties, which help protect the pancreas. Specifically, quercetin may protect pancreatic islet β cells from oxidation-induced apoptosis by upregulating the sirtuin 3 (Sirt3) protein [33]. Sirt3 regulates mitochondrial antioxidant defense [34]. Additionally, quercetin may reduce ferroptosis, an iron-dependent form of programmed cell death that contributes to pancreatic cell destruction [7]. Thus, quercetin may help preserve the viability of islet β cells and restore their ability to produce and secrete insulin.

In diabetic patients with insulin resistance, elevated blood insulin levels are typically observed due to the reduced responsiveness of cells to insulin [54,55]. In rats with fructose-induced insulin resistance, the oral administration of quercetin at a dose of 50 mg/kg for 36 days improved insulin sensitivity, as indicated by a reduced homeostasis model assessment of insulin resistance (HOMA-IR) index and a decreased area under the curve in the oral glucose tolerance test [47]. This improvement in insulin sensitivity by quercetin is believed to be associated with the upregulation of SIRT1, which enhances glucose uptake from the bloodstream, likely through the upregulation of glucose transporter type 4 (GLUT4) [56]. As a result, the demand on the pancreas to produce insulin is reduced. More than 80% of glucose uptake occurs via insulin-sensitive skeletal muscles, and quercetin has been shown to promote GLUT4 translocation in skeletal muscle cells, the most downstream factor in the insulin signaling cascade [57,58]. When combined with metformin, an oral hypoglycemic agent, quercetin shows a synergistic effect in reducing blood glucose levels in diabetic rats [37,59]. Metformin works by reducing hepatic gluconeogenesis, improving insulin sensitivity, and decreasing intestinal glucose absorption [60], suggesting that quercetin may exert additional mechanisms beyond those of metformin.

Hyperglycemia, particularly in the context of insulin resistance, impairs cholesterol metabolism in the liver by inducing excessive cholesterol biosynthesis, leading to dyslipidemia [61]. The beneficial effects of quercetin on the blood lipid profile of diabetic animals have been demonstrated in various studies (Table 2). However, the combination of quercetin and metformin does not provide additional benefits in improving blood lipid levels [37]. Quercetin reduces blood cholesterol by downregulating the expression of proprotein convertase subtilisin/kexin type 9 (PCSK9) [62], an enzyme that binds to hepatic low-density lipoprotein (LDL) receptors and promotes their degradation. By reducing PCSK9 levels, quercetin helps maintain the availability of LDL receptors, thereby enhancing LDL removal from the bloodstream [63]. Additionally, quercetin enhances the expression of liver X receptor α (LXRα), a regulator of cholesterol transport, which subsequently activates ATP-binding cassette transporter A1 (ABCA1) [62]. ABCA1 facilitates the efflux of cholesterol to apolipoprotein AI (ApoA1), promoting high-density lipoprotein (HDL) formation and aiding in cholesterol removal by the liver [64]. Quercetin also downregulates the expression of cluster of differentiation 36 (CD36) in diabetic hearts, a membrane glycoprotein that facilitates the uptake of free fatty acids into the sarcolemma and their transport to endosomal compartments in cardiomyocytes [60]. The upregulation of CD36 mediates fatty acid sensing through the activation of peroxisome proliferator-activated receptor-γ (PPARγ) [65], which increases lipid storage and contributes to dilated cardiomyopathy in mice [66]. However, quercetin was found to downregulate CD36 expression, which in turn decreased heart cholesterol levels in high-cholesterol diet-induced diabetic rats [38]. Emerging research on the CD36-PPARγ–fatty acid transporter protein 1 (FATP1) pathway suggests that PPARγ activation may be beneficial by suppressing FATP1, thus reducing free fatty acid uptake from the extracellular space [67]. Therefore, modulating CD36 and/or activating PPARγ may help normalize lipid uptake and prevent free fatty acid overload, which otherwise contributes to lipotoxicity, insulin resistance, and an increased risk of diabetic atherosclerosis. Further studies are needed to investigate quercetin’s effects on the CD36-PPARγ-FATP1 pathway, as its influence on FATP1 remains unclear.

The combined effects of quercetin lead to a reduction in blood cholesterol levels, which in turn decreases the risk of diabetic cardiomyopathy. Despite the aforementioned positive findings, quercetin showed no effect on blood cholesterol levels in Zahedi et al. [69], Ferenczyova et al. [41], and Boťanská et al. [39]. The dosage and duration of the treatment may be the key factors affecting the clinical effectiveness of quercetin, which warrant further clinical trials to confirm the findings. Clinical trials with a more robust design, a larger sample size, and varying doses should be conducted in human subjects to reach a definitive conclusion about quercetin’s effectiveness against hyperlipidemia. Meanwhile, the lack of effects observed in Ferenczyova et al. [41] and Boťanská et al. [39] may be attributed to factors such as aging (1-year-old rats) and obesity, both of which can impair lipid metabolism in diabetic rats.

## 4. Effects of Quercetin on Myocardial Oxidative Stress and Inflammation

The excessive production of hyperglycemia-induced reactive oxygen species (ROS) is widely recognized as a key factor in the development of diabetic cardiomyopathy. This has been confirmed through in vitro studies involving various cell types exposed to high glucose [11,18,70].

**Figure 3:**
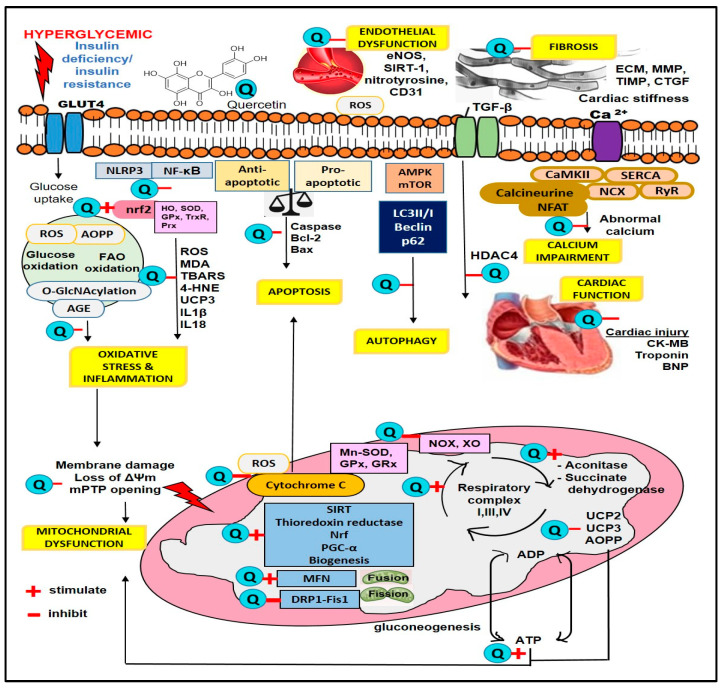
Sites of quercetin action in hyperglycemia. 4-HNE, 4-Hydroxynonenal; ADP, adenosine diphosphate; AGE, advanced glycation end product; AOPPs, advanced oxidation protein products; ATP, adenosine triphosphate; CK-MB, creatine kinase MB; DRP-1, dynamin-related protein-1; ECM, extracellular matrix; eNOS, endothelial nitric oxide synthase; Fis1, fission 1 protein; GLUT4, glucose transporter 4; GPx, glutathione peroxidase; GRx, glutathione reductase; HDAC4, histone deacetylase 4; HO, heme oxygenase; IL-1β, interleukin-1β; MDA, malondialdehyde; MFN, mitofusin; MMP, matrix metalloproteinase; Mn-SOD, manganese superoxide dismutase; NF-κB, nuclear factor-kappa B; NLRP3, nucleotide-binding oligomerization domain-like receptor family pyrin domain-containing 3; NOX, NADPH oxidase; Nrf, nuclear factor erythroid 2-related factor; TBARS, thiobarbituric acid reactive substance; PGC-α, peroxisome proliferator-activated receptor gamma coactivator 1-alpha; ROS, reactive oxygen species; SIRT, sirtuin; SOD, superoxide dismutase; TGF-β, transforming growth factor beta; UCP, uncoupling protein; XO, xanthine oxidase.

In experimental diabetic animals, quercetin at doses of 5–160 mg/kg for 4–12 weeks demonstrated protective effects against the elevation of myocardial ROS and lipid peroxidation products [10,11,18,38,47,48,49] (Table 3). This protection was accompanied by an increase in the activity of key antioxidant enzymes, including superoxide dismutase (SOD), glutathione peroxidase (GPx), and catalase [11,18,35,38,39,47,49]. SOD, as a primary defense mechanism in cells, neutralizes superoxide—a type of ROS—by converting it into oxygen and hydrogen peroxide [16]. The resulting hydrogen peroxide is then decomposed into oxygen and water by glutathione peroxidase and catalase [71]. The findings suggest that quercetin abolishes the generation of myocardial ROS by activating antioxidant defense mechanisms in diabetic cardiomyopathy (Figure 3).

However, two studies reported a reduction in antioxidant enzyme activity in streptozotocin-induced diabetic rats treated with quercetin [40,66]. In Coldiron et al. [40], diabetes did not affect antioxidant enzyme activity despite elevated lipid peroxidation, but quercetin treatment reduced enzyme activity, possibly indicating that quercetin’s antioxidant effects were sufficient to manage oxidative stress without needing to boost endogenous defenses. Conversely, in Stechyshyn and Pavliuk [68], diabetic rats exhibited increased antioxidant enzyme activity after 2 weeks of diabetes induction, and quercetin treatment normalized these changes. This suggests that early in diabetes (2 weeks), the body may adapt to rising ROS by enhancing antioxidant defenses, while in later stages (longer than 4 weeks), oxidative stress may exhaust this capacity.

An interesting finding was also reported by Gorbenko et al. [43], which showed quercetin (10 mg/kg and 50 mg/kg/d for 8 weeks) dose-dependently decreased the myocardial mitochondrial levels of advance oxidation protein products (mtAOPPs)—a relatively novel marker of oxidative damage—in diabetic rats. Associated with this reduction were the normalized activities of mitochondrial antioxidant enzymes, including manganese SOD (mtMn-SOD), GPx, and glutathione reductase (mtGRx). The contradictory trends in antioxidant enzyme activity reported in this study compared to the findings of Roslan et al. [49], Castillo et al. [38], Kushwah and Gupta [47], and Chang et al. [18] may be explained by negative feedback mechanisms aimed at maintaining redox homeostasis. Quercetin is known to upregulate antioxidant enzymes in environments with massive ROS generation. However, the observed inhibition in the quercetin-treated group is speculated to be due to its ability to prevent the hyperproduction of ROS. The reduction in mtAOPPs is attributed to quercetin’s inhibition of major ROS-generating systems, specifically NADPH oxidase (NOX) and xanthine oxidase (XO), as evidenced by the reduced levels of NOX and XO in the quercetin-treated group [41].

Quercetin enhances myocardial antioxidant defenses by upregulating nuclear factor erythroid 2-related factor 2 (Nrf2), a crucial transcription factor in the cellular response to oxidative stress [11,35,38,53]. Under normal conditions, Nrf2 is bound to its suppressor, Kelch-like ECH-associated protein 1 (Keap1). During oxidative stress, Nrf2 dissociates from Keap1 and translocates to the nucleus, where it activates genes containing the antioxidant response element (ARE). This activation triggers the production of various antioxidant enzymes, including heme oxygenase, glutamate cysteine ligase (GCLC), SOD, GPx, catalase, thioredoxin reductase (TrxR), and peroxiredoxin (Prx) [11,74]. Consequently, the activation of Nrf2 by quercetin leads to the increased expression of these antioxidant enzymes in diabetic cardiomyopathy, as observed by various studies (Table 3).

The presence of 8-isoprostane indicates oxidative damage to cell membrane lipids. Lipid peroxidation not only disrupts membrane integrity but also produces secondary products like malondialdehyde (MDA) and 4-hydroxynonenal (4-HNE), which can further damage proteins and DNA, contributing to disease progression [75,76]. Moreover, elevated ROS production in mitochondria triggers mitochondrial uncoupling, primarily mediated by uncoupling proteins (UCPs) 2 and 3. Their excessive activation reduces ATP production, thereby exacerbating cardiac dysfunction in the energy-demanding myocardium [77]. A reduction in 8-isoprostane, MDA, 4-HNE, and thiobarbituric acid reactive substances (TBARSs)—markers of lipid peroxidation as well as UCPs—was observed in diabetic rats and HL-1 cells treated with quercetin [35,38,72].

Besides contributing to oxidative stress, ROS can also trigger inflammatory reactions, leading to an activation of the nuclear factor-kappa B (NF-κB) inflammatory pathway. Following activation, which is regulated by the inhibitor of the NF-κB kinase (IKKα and IKKβ), the nuclear translocation of NF-κB occurs. Quercetin suppresses the activation of the myocardial NF-κB pathway in experimental diabetic animals, inhibiting inflammatory factors, such as tumor necrosis factor-alpha (TNF-α) and interleukin 1β and 6 (IL-1β and IL-6) [49,51] (Table 3). Additionally, the aberrant activation of myeloperoxidase (MPO) and the nucleotide-binding oligomerization domain-like receptor family pyrin domain-containing 3 (NLRP3) may also trigger inflammation. Studies have demonstrated that quercetin reduces MPO levels [73] and downregulates NLRP3 expression [10,11,18], thereby suppressing inflammation and oxidative stress. Collectively, as noted above, quercetin inhibits cardiac oxidative stress and inflammation via the modulation of Nrf2, NF-κB, and NLRP3 signaling pathways, increasing the antioxidant capacity of the diabetic myocardium. However, its detailed molecular mechanisms and the sequence of events involving quercetin’s role in oxidative stress and inflammation are still lacking.

## 5. Effects of Quercetin on Heart Fibrosis

Heart fibrosis develops as a reparative response to chronic inflammation, characterized by the excessive production of extracellular matrix components, including collagen, surpassing its degradation. This leads to tissue stiffening [8]. Quercetin protects against myocardial fibrosis in diabetic animals by reducing collagen gene expression and its subsequent accumulation [8,10,11] (Table 4). This protective effect of quercetin is attributed to the downregulation of key growth factors, specifically connective tissue growth factor (CTGF) and transforming growth factor-β1 (TGFβ1), which are major drivers of fibrosis [10,51] (Figure 3). The reduced expression of these growth factors aligns with their regulation by histone deacetylase 4 (HDAC4) and myocyte enhancer factor 2 (MEF2) [8].

The antifibrotic effects of quercetin have not been extensively studied. To date, only one study [39] has examined the effects of quercetin on proteins involved in extracellular matrix degradation, specifically matrix metalloproteinase-2 (MMP-2) and its inhibitor, tissue inhibitor of metalloproteinase-2 (TIMP-2), in a diabetes model. This study found that in obese diabetic rats, the protein levels of 63 kDa MMP-2 and the activity of 72 kDa MMP-2 (inactive) were increased. Since 63 kDa MMP-2 may represent the active form of MMP-2 [78], the total MMP-2 activity would be elevated. Although quercetin was reported to reduce the diabetes-induced expression of 63 kDa MMP-2, the expression and activity of 72kDa MMP-2 were not significantly affected [39], creating uncertainty regarding the significant effects of quercetin on modulating MMP-2. MMP-2 is responsible for collagen degradation, and its activity is regulated by TIMP-2. Furthermore, the role of myofibroblasts—the primary producers of collagen—in quercetin’s antifibrotic mechanisms remains unexplored. HDAC4 indirectly influences myofibroblast activation by modulating gene expression, which promotes the activity of growth factors that facilitate the differentiation of fibroblasts into myofibroblasts [79].

Taken together, quercetin may have a beneficial effect on myocardial fibrosis by reducing its development and preventing further complications in diabetic patients, such as heart failure and arrhythmias. However, further research is required to fully elucidate the mechanisms through which quercetin influences myocardial fibrosis and to confirm its therapeutic potential in slowing the progression of diabetic heart disease.

## 6. Effects of Quercetin on Myocardial Apoptosis and Autophagy

Apoptosis, a form of programmed cell death, is a crucial process in the progression of diabetic cardiomyopathy, with mitochondria serving as the central regulators of cellular apoptosis. During this process, the activity of major pro-apoptotic markers, such as caspase-3 and caspase-9, becomes elevated. Quercetin treatment at doses ranging from 10 to 100 mg/kg over a period of 4 to 24 weeks has been shown to reduce caspase activity in the myocardium of diabetic rats [9,10,11,35,36,48,49] (Table 5). Furthermore, quercetin significantly reduces the terminal deoxynucleotidyl transferase dUTP nick-end labeling (TUNEL)-stained area and apoptosis rate in cardiomyocytes [9,18,35].

Quercetin protects against apoptosis by modulating the balance between the anti-apoptotic protein B-cell lymphoma-2 (Bcl-2) and the pro-apoptotic protein Bcl-2-associated X protein (Bax). It enhances Bcl-2 expression while decreasing Bax levels [9,35,49] (Figure 3). These proteins play opposite roles in regulating apoptosis by controlling mitochondrial membrane permeability [80]. Under cellular stress, Bax is activated, promoting mitochondrial membrane permeabilization, which damages the outer membrane and leads to the release of cytochrome c [80]. Quercetin reduces cytochrome c release [35,48].

These findings suggest that quercetin exerts anti-apoptotic effects by balancing pro-apoptotic and anti-apoptotic events, likely due to its antioxidant properties. ROS are known to cause mitochondrial damage [42]. However, many aspects of apoptosis in relation to quercetin’s effects remain unexplored. For example, in the extrinsic apoptosis pathway, cell surface death receptors like the Fas receptor interact with their ligands, such as FasL, leading to the activation of caspases and the initiation of apoptosis [80]. Additionally, endoplasmic reticulum stress can trigger apoptosis [81], and quercetin may influence this process as well. Indeed, further studies, such as in vivo and in vitro experiments, need to be conducted to confirm this mitigation.

Apoptosis is a process of controlled cellular destruction that leads to cell death, whereas autophagy primarily promotes cell survival by removing and salvaging damaged cellular components [82]. However, only one study has investigated the effects of quercetin on autophagy in diabetic cardiomyopathy. In this study, quercetin was found to restore autophagy in the myocardium of streptozotocin-induced diabetic rats by upregulating the expression of autophagy-related proteins microtubule-associated protein light chain 3II/I (LC3II/I) and Beclin-1 [9] (Table 6). LC3II/I is crucial for initiating autophagosome formation, which sequesters cellular waste, while Beclin-1 is crucial in autophagosome maturation [83]. Additionally, quercetin downregulated p62 protein levels [9], a marker of autophagic flux that tags damaged cellular constituents for degradation [84]. These changes collectively indicate that quercetin enhances autophagy by promoting autophagosome formation and the clearance of cellular debris. The increased expression of LC3II/I and Beclin-1, combined with the reduced p62 levels by quercetin, suggests a more efficient autophagic process, ultimately contributing to improved myocardial health in diabetic conditions. Comparable results were observed in H9c2 cells exposed to high glucose conditions [9].

Moreover, quercetin enhances the expression of phosphorylated adenosine mono-phosphate-activated protein kinase (p-AMPK)—an enzyme that regulates cellular energy—while inhibiting the expression of the phosphorylated mechanistic target of rapamycin (p-mTOR)—a protein that suppresses autophagosome formation [85]—in H9c2 cardiomyocytes exposed to high glucose [9]. Taken together, the compound promotes autophagy, which is often linked to reduced apoptosis [86]. This relationship is consistent with the observed effects of the compound. The dissociation of the Bcl-2–Beclin-1 complex by activated AMPKs should be studied further, as it could bidirectionally impact the effectiveness of quercetin in protecting against diabetic cardiomyopathy in the future.

## 7. Effects of Quercetin on Heart Mitochondrial Function and Energy Metabolism

Mitochondria are highly dynamic organelles known as the ‘powerhouses’ of the cells, supplying energy in the form of ATP. Damage to these organelles can disrupt their function, potentially leading to cardiac dysfunction, including conditions such as cardiomyopathy. Mitochondrial dysfunction is considered a driving force in the pathogenesis of diabetic cardiomyopathy [87]. Chang et al. [18] reported that high-glucose-stimulated HL-1 cells exhibited serious disorders in mitochondrial energy metabolism. Quercetin treatment (150 mg/L) reversed this deleterious effect by restoring cardiac energy metabolism, as evidenced by a marked increase in basal respiration, maximal respiration, and the respiratory reserve of mitochondria, as well as a decreased opening of permeability transition pore (mPTP) activity, thus increasing ATP production, suggesting the beneficial effect of quercetin on mitochondrial function capacity and bioenergetics (Table 7). ATP is primarily generated from the oxidative phosphorylation process involving mitochondrial respiratory complexes I, III, and IV [88]. A high-glucose microenvironment impairs mitochondrial function, leading to a decrease in the efficiency and activity of mitochondrial respiratory complexes due to oxidative stress, protein glycation, and reduced biogenesis, which alters mitochondrial dynamics, consequently reducing ATP production. Quercetin has been demonstrated to restore the activities of these complexes [18], thus improving oxidative phosphorylation (Figure 3).

Chang et al. [18] further explored the possible mechanism of the protective effects of quercetin on mitochondrial energy metabolism in high-glucose-stimulated HL-1 cells. The effect of quercetin on sirtuin 5 (*SIRT5*) was investigated in HL-1 myocytes incubated in high glucose and treated with lipofectamine RNAiMAX stably transfected with si-SIRT5 for silencing *SIRT5*. This treatment abolished the protective effects of quercetin on the aforementioned parameters and inhibited NADPH-dependent isocitrate dehydrogenase 2 (*IDH2*) mRNA transcription and IDH2 succinylation levels, indicating that quercetin im-proves mitochondrial activity by regulating the *SIRT5*-mediated desuccinylation of IDH2, thus maintaining mitochondrial homeostasis.

The generation of new mitochondria by mitochondrial biogenesis is regulated by transcription factors, including peroxisome proliferator-activated receptor gamma coactivator 1-alpha (PGC-1α), nuclear respiratory factor 1 (NRF1), and mitochondrial transcription factor A (Tfam) [89]. Mitochondrial fusion and fission are dynamic processes that regulate their shape, size, number, and function. The key protein involved in mitochondrial fusion is mitofusin (Mfn), while dynamin-related protein 1 (DRP1) is central to mitochondrial fission. Mitochondrial fission 1 protein (Fis1) is one of the proteins that act as receptors to regulate Drp1 activity during mitochondrial fission. Quercetin was found to upregulate the transcriptional levels of *PGC-1α*, *Tfam*, and *Mfn*, as well as downregulate the *Drp1* and *Fis1* genes, leading to an improvement in mitochondrial DNA content in HL-1 cells [18].

In an animal study, post-treatment with quercetin (50 mg/kg/day for 8 weeks) normalized the metabolic activity of heart mitochondria, as evidenced by increased levels of thioredoxin reductase, aconitase, and succinate dehydrogenase, along with reduced levels of advanced oxidation protein products (AOPPs) and a decreased rate of calcium-dependent mitochondrial swelling in streptozotocin-induced diabetic rats fed a high-calorie diet [42]. Hyperglycemia leads to excessive ROS production and AOPP formation, with AOPPs serving as a novel marker of mitochondrial oxidative damage. Mitochondrial thioredoxin reductase plays a key role in defending against oxidative stress [90]. Aconitase and succinate dehydrogenase, essential enzymes in the tricarboxylic acid cycle, are crucial for mitochondrial homeostasis. Their impairment disrupts bioenergetics, leading to citrate accumulation and a slowdown in oxidative phosphorylation, which impacts cellular energy balance [42,91,92]. The protective effects of quercetin on heart mitochondria suggest that it may help restore mitochondrial health by decreasing oxidative stress and improving mitochondrial bioenergetics, ultimately enhancing heart function.

UCPs are key pathogenic determinants in cardiovascular and metabolic diseases, with distinct roles in metabolism and energy regulation [93]. Quercetin treatment (50 mg/kg) in streptozotocin-induced diabetic Wistar rats for 8 weeks, as well as in rats fed a high-cholesterol diet for 4 weeks, significantly reduced UCP-2 and UCP-3, resulting in increased ATP production [35,38]. The reduction in these pathogenic markers by quercetin is linked to the upregulation of the transcription factor *PGC-1α* and the downregulation of PPAR-γ, which help prevent bioenergetic alterations and improve contractile function [38].

Altogether, these studies suggest that quercetin can preserve mitochondrial function and improve energy metabolism under hyperglycemic conditions, leading to enhanced myocardial contractile performance. This likely occurs through the modulation of key factors such as SIRT5, PGC-1α, Tfam, Mfn, PPARγ, DRP-1, mitochondrial thioredoxin reductase activity, and tricarboxylic acid cycle key enzymes, all of which play vital roles in mitochondrial quality control and homeostasis. Future studies could explore the effect of quercetin on the nuclear respiratory factor (NRF), the mitochondrial fission factor (MFF), and mitochondrial dynamics 49 (MiD49) and 51 (MiD51), which are involved in mitochondrial bioenergetics and fission. Additionally, its impact on mitochondrial mitophagy (selective degradation of damaged or dysfunctional mitochondria through the autophagy pathway), proteostasis (protein quality control), and mitochondrial DNA repair warrants further investigation.

## 8. Effects of Quercetin on Cardiac Structure

Diabetes-induced cardiomyopathy leads to abnormal myocardial structure, impairing both systolic and diastolic heart functions. This triggers compensatory mechanisms, particularly mitochondrial biogenesis, as well as interstitial and perivascular fibrosis, which eventually result in changes to left ventricular wall geometry. Structurally, quercetin treatment in rats has been shown to reduce left ventricular mass and relative overall wall thickness [7,8,38]. Additionally, quercetin administration decreased the heart weight-to-body weight ratio and cardiac somatic index in rat models of hyperglycemia induced by a high-cholesterol diet and streptozotocin [38,42,52], as well as in streptozotocin-induced diabetic mice fed a high-calorie diet [9] (Table 8). Histologically, quercetin treatment in diabetic rats has improved cardiomyocyte structure, showing a regular alignment of heart muscle fibers with branching and anastomosis resembling normal heart tissue. In contrast, diabetic rats exhibited disorganized fibers with diminished striation and a loss of anastomoses. The cardioprotective effects of quercetin were further supported by a reduction in interventricular septal thickness at diastole (IVSd) and in left ventricular posterior wall thickness at end-diastole (LVPWd), as well as the normalization of the left ventricular internal diameter at end-diastole (LVIDd) [8]. Moreover, quercetin significantly improved left ventricular remodeling, as evidenced by a reduction in left ventricular end-diastolic diameter (LVEDD) and left ventricular end-systolic diameter (LVESD), leading to improved echocardiographic cardiac geometry [9].

Treatment with quercetin significantly reduced cardiac injury biomarkers, as evidenced by the decreased release of myocardial injury markers such as creatine kinase, creatine kinase MB, and cardiac troponin [10,35,44,47,49] (Table 8). Additionally, quercetin lowered key cardiac hypertrophy mediators, including B-type natriuretic peptide (BNP), GATA-binding protein 4 (GATA4), and serum response factor [8,11,51]. In studies involving myocardial ischemia/reperfusion (I/R) injury, the intraperitoneal administration of 5 and 10 mg/kg quercetin 10 min prior to I/R induction reduced myocardial infarct size in streptozotocin-induced diabetic rats [72,94]. Similarly, the oral administration of 25 mg/kg quercetin for 30 days in streptozotocin-induced diabetic rats reduced myocardial infarct size, as well as vacuolization, congestion, infiltration, and myofibril loss in myocardial tissue [36]. Quercetin (20 mg/kg/day administered in biscuits for 6 weeks) did not reduce infarct size in the I/R-induced heart injury of older and obese Zucker diabetic fatty rats (6–12 months old) [41]. This lack of effect may be attributed to the impaired activation of the phosphoinositide 3-kinase/protein kinase B (PI3K/Akt) pathway due to aging and prolonged diabetes progression (with higher glycemia levels), potentially nullifying quercetin’s cardioprotective effects in older rats [41]. The PI3K/Akt signaling pathway plays a role in regulating cell growth, oxidative stress, inflammation, and fibrosis [76,94]. It is also a component of the Reperfusion Injury Salvage Kinase (RISK) pathway, a key cardioprotective signaling cascade [41].

These findings suggest that multiple prohypertrophic signaling pathways are in-volved in quercetin’s effects, including transcription factors that mediate structural proteins and hypertrophic growth. Quercetin protects heart structure through its modulation of the MEF2/HDAC4/extracellular signal-regulated kinase 5 (ERK5) pathway [8]. The inhibition of MEF2, HDAC4, and ERK5 activities prevents adverse cardiac remodeling, which is beneficial in protecting against diabetic cardiomyopathy. The cardioprotective effects of quercetin are likely due to reduced oxidative stress, moderate increases in antioxidant reserves, and improved mitochondrial biogenesis and cardiac fibrosis. However, further clinical studies are needed to fully understand how quercetin improves cardiac structure in humans.

## 9. Effects of Quercetin on Cardiac Function

Diabetic cardiomyopathy initially presents as diastolic dysfunction and progresses to systolic dysfunction, accompanied by altered ventricular relaxation, leading to impaired left ventricular function [3]. Diabetic animal models often show evident diastolic dysfunction with or without systolic impairment. Restoring these parameters is essential for maintaining normal cardiac performance. In a clinical study, quercetin supplementation (500 mg/kg for 10 weeks) in women with type 2 diabetes mellitus (n = 72) reduced systolic blood pressure but had no significant effect on diastolic blood pressure, suggesting potential blood pressure-lowering properties [69]. However, this is the only clinical study available reporting quercetin’s impact on cardiovascular function in diabetic subjects, highlighting the need for further studies with more robust designs to validate its benefits.

In diabetic animal models, quercetin has shown beneficial effects on heart function, including increases in ejection fraction and fractional shortening [10,46]. It also restores mean arterial blood pressure (MABP), left ventricular systolic pressure (LVSP), left ventricular end-diastolic pressure (LVEDP), and contractility indices, such as maximal rates of pressure fall (-dP/dt_max_)/LVSP, to their normal levels [35,49]. Quercetin has been shown to prevent high-cholesterol diet-induced diastolic dysfunction in diabetic rats, as evidenced by a reduction in atrial filling velocity (A wave) and an increase in the ratio of peak velocity of early to late filling of mitral inflow (E/A) [38] (Table 9). In contrast, Bartosova et al. [8] found that quercetin selectively decreased the E/A ratio and improved diastolic dysfunction in Zucker diabetic fatty rats. This discrepancy could be due to the fact that before treatment, diabetic animals had an elevated E/A ratio compared to their lean controls, which quercetin selectively reversed. This may explain the divergent findings. Additionally, the E/A ratio, while a common diagnostic marker for diastolic function, is less sensitive and can be difficult to interpret in clinical settings. The ratio of early diastolic mitral inflow velocity to early diastolic mitral annular velocity (E/E’) is considered a more sensitive indicator of diastolic function [95]; however, it was not assessed in these studies.

The heart’s electrical activity coordinates the heartbeat by propagating electrical im-pulses through the myocardium, resulting in P, QRS (wave complex), and T waves. The T wave represents ventricular repolarization, and the T-P interval is the time between the end of the T wave and the start of the next P wave. The T peak-to-T end interval measures the time from the peak to the end of the T wave [96]. The QT interval reflects ventricular repolarization, with its prolongation in diabetic patients being associated with an increased risk of myocardial infarction. Shortened R-R intervals and elevated heart rates in diabetic cardiomyopathy lead to accelerated rhythms and sinus tachycardia [45]. Quercetin (50 mg/kg) mitigated heart rate-corrected QT interval (QTc) prolongation [35], and similarly prolonged R-R intervals in streptozotocin-induced diabetic rats, preventing the progression of tachycardia [45]. At a lower dose (10 mg/kg), quercetin did not affect the shortening of R-R intervals. Regardless of its dose, quercetin shortened the T wave, suggesting it positively impacts ventricular repolarization, possibly through the inhibition of ischemic processes in the myocardium [45].

Abnormal electrophysiological phenomena that impair heart function have been observed in streptozotocin-induced diabetic rats. Two-pore domain weak inwardly rectifying potassium (TWIK)-related acid-sensitive potassium (TASK-1) channels, predominantly expressed in the atria, regulate various electrophysiological processes, including action potential duration. Several clinically relevant antiarrhythmic drugs—such as propafenone, mexiletine, lidocaine, and quinidine—act by inhibiting homodimeric TASK-1 channels at physiological or subtherapeutic concentrations [97]. Similarly, quercetin treatment has been shown to improve TASK-1 channel dysfunction in the ventricular myocytes of diabetic rats. Specifically, quercetin increased TASK-1 current density and shortened the action potential duration at 50% and 90% repolarization (APD50 and APD90) in diabetic myocardium, highlighting its potential as a therapeutic agent for managing arrhythmias [10].

Moreover, quercetin has been shown to improve the functional state of autonomic regulation in diabetic heart activity, as evidenced by the normalization of heart rate variability parameters such as the R-R interval, vegetative equilibrium index (VEI), index of tension (IT), vegetative rhythm indicator (VRI), and regulatory process adequacy index (RPAI) [52]. This results in a balanced interaction between sympathetic and parasympathetic nervous system activity [98]. Additionally, Stechyshyn et al. [52] compared the effects of water-soluble quercetin and liposomal quercetin on heart rhythm variability and found that liposomal quercetin exhibited superior efficacy in diabetic rats. This enhanced effect is attributed to the lecithin content in liposomal quercetin, which helps maintain a more stable and prolonged effect, protecting the drug molecule from degradation.

Based on these studies, quercetin shows promise in improving heart function and managing arrhythmia. However, further research is needed, particularly in clinical settings, to establish its impact on left ventricular function. The effects of quercetin on right ventricular function remain unexplored and warrant further investigation.

## 10. Effects of Quercetin on Myocardial Calcium Regulation

Ventricular shortening is the contraction of the ventricles during systole, critical for effective blood circulation. Myocardial contractility depends on calcium regulation, and disruptions in calcium handling contribute to diabetic cardiomyopathy [98]. Quercetin-3-O-glucoside reduces the time to peak (TPK) shortening in streptozotocin-induced diabetic rats, aligning with control group levels, indicating improved cardiac contractility [99] (Table 10). Additionally, quercetin-3-O-glucoside exhibits negative inotropic effects by decreasing the amplitude of shortening, calcium transient, and myofilament sensitivity to calcium without affecting calcium release from the sarcoplasmic reticulum. These effects may benefit diabetic patients at risk of angina, myocardial infarction, and heart failure [99].

Sarcoplasmic/endoplasmic reticulum calcium ATPase (SERCA) is responsible for the uptake of calcium into cells and the sarcoplasmic reticulum, playing a crucial role in muscle relaxation and calcium homeostasis [102]. In contrast, the sodium–calcium exchanger (NCX) regulates the exchange of intracellular sodium for calcium, helping to maintain calcium balance in cardiomyocytes [103]. Together, these two proteins coordinate calcium dynamics, which are essential for normal cardiac function, including both contraction and relaxation. Diastolic dysfunction, a hallmark of diabetic cardiomyopathy, presents itself as a promising therapeutic target for improving myocardial relaxation. Quercetin has been shown to exert a positive lusitropic effect by accelerating myocardial relaxation in the diabetic myocardium through the activation of SERCA without affecting the NCX [101]. This finding highlights quercetin’s potential for restoring normal diastolic function in the myocardium.

Calcium/calmodulin-dependent protein kinase II (CaMKII) enhances calcium uptake by promoting SERCA activity [104]. Research indicates that the CaMKII pathway and the calcium–calcineurin cascade, along with the downstream target nuclear factor of activated T cells (NFAT), play a critical role in calcium regulation under hypertrophic conditions [105]. In a study by Bartosova et al. [8], quercetin treatment reduced the expression of calcineurin A, NFAT3, and GATA4 in diabetic rats, but did not affect CaMKII or pThr286-CaMKII levels. This suggests that quercetin improves myocardial contractility through the modulation of the calcineurin-NFAT3 pathway (Figure 3).

Collectively, these findings indicate that quercetin helps maintain cardiac calcium homeostasis and membrane integrity through the SERCA/calcineurin-NFAT3 pathway, ensuring normal cardiac contractile function under diabetic conditions. The effects of quercetin on other calcium regulation-related proteins, such as sodium–potassium ATPase (Na^+^/K^+^-ATPase), which indirectly influences calcium homeostasis in cardiomyocytes by regulating the sodium and potassium ion balance, as well as ryanodine receptor type 2 (RyR2), responsible for calcium release, and phospholamban, which regulates calcium uptake, remain to be further investigated.

## 11. Effects of Quercetin on Endothelial Dysfunction

As previously discussed, quercetin significantly improves dyslipidemia in diabetic rats, including reductions in the apolipoprotein B (ApoB)/A1 ratio, total cholesterol, triglycerides, and LDL-to-HDL ratios. These effects are particularly pertinent given that endothelial dysfunction in coronary microvessels plays a critical role in the onset and progression of diabetic cardiomyopathy by contributing to microvascular damage, impaired myocardial perfusion, metabolic disturbances, and diastolic dysfunction [106,107]. Apolipoproteins, particularly ApoB and ApoA1, are essential for lipid metabolism and are closely linked to endothelial dysfunction, a hallmark of atherosclerosis and cardiovascular disease [108]. Notably, quercetin intake at doses of 50 and 150 mg/kg reduced the ApoB/A1 ratio by over 20%, improved the lipid profile, and decreased VLDL-triglyceride production in glucocorticoid-induced dyslipidemic rats [109] and diet-induced obesity mouse models [110]. These findings highlight quercetin’s potential to protect endothelial function by improving lipid and apolipoprotein profiles.

To the best of our knowledge, only one study has investigated the effects of quercetin on endothelial dysfunction in a diabetic model. Chellian et al. [37] demonstrated that the oral administration of quercetin at 10 mg/kg for 30 days reversed hyperglycemia-induced endothelial dysfunction in aortic rings isolated from streptozotocin-nicotinamide-induced diabetic rats. Quercetin improved acetylcholine-induced endothelium-dependent relaxation, enhanced sodium nitroprusside-induced endothelium-independent relaxation, and reduced phenylephrine-induced contraction in the diabetic rats, with no similar effects observed in the control group. These findings suggest that quercetin promotes vasorelaxation in diabetes through multiple mechanisms, including increased vascular sensitivity to nitric oxide, activation of muscarinic receptors, and other alternative pathways, thereby protecting against endothelial dysfunction.

Hyperglycemia induces the production of ROS and AGEs, leading to the downregulation of endothelial nitric oxide synthase (eNOS) and reduced nitric oxide (NO) bioavailability [106]. Under conditions of oxidative stress and inflammation, markers of endothelial damage, such as vascular cell adhesion molecule-1 (VCAM-1), cluster of differentiation 31 (CD31), and nitrotyrosine, are elevated. Quercetin treatment was shown to upregulate SIRT1 and eNOS expression while reducing nitrotyrosine, CD31, and VCAM-1 levels in the abdominal and thoracic aortae of diabetic rats [37]. This suggests that quercetin activates SIRT1, leading to eNOS phosphorylation and enhanced NO bioavailability, thereby improving vascular function in diabetic rats. Furthermore, the combination of quercetin and metformin demonstrated superior anti-diabetic effects compared to either agent alone, underscoring its therapeutic potential in managing dyslipidemia and endothelial dysfunction—key contributors to the progression of diabetic cardiomyopathy [37,59].

## 12. Meta-Analysis of the Clinical Impact of Quercetin on Cardiovascular Health

Table 11 summarizes a meta-analysis of human intervention studies on quercetin supplementation in patients with metabolic syndrome [110,111,112,113,114,115], showing mixed results for cardiovascular parameters. However, longer treatment durations exceeding 8 weeks show more pronounced beneficial effects of quercetin [110]. Further research is needed to confirm the clinical significance of these findings and explore quercetin’s potential for therapeutic applications.

## 13. Conclusions and Future Directions for Research

Quercetin has emerged as a compelling candidate for the management of diabetic cardiomyopathy, demonstrating the ability to alleviate disease progression through its regulation of multiple pathways, including glucose and lipid homeostasis, oxidative stress, inflammation, fibrosis, apoptosis, autophagy, mitochondrial function, calcium regulation, and endothelial dysfunction. As shown in Figure 3, quercetin targets several key molecules and signaling cascades, which collectively improve the underlying mechanisms of diabetic cardiomyopathy. Although multitargeted therapeutic agents like quercetin are generally considered less potent than monotargeted drugs, their broader action can lead to greater efficacy, synergistic effects, an improved safety profile, and a reduced risk of drug resistance and interactions.

Moreover, quercetin’s potential to target O-GlcNAcylation and AGEs presents promising strategies for mitigating hyperglycemia-induced cardiac damage. However, further research is required to validate these targets’ therapeutic potential, as the current evidence primarily stems from cellular and animal studies, with some inconsistent or conflicting results. Therefore, clinical trials are essential to evaluate the true effects of quercetin in patients with diabetic cardiomyopathy.

Additionally, exploring the combination of quercetin with metformin could yield valuable insights, as this combination has demonstrated synergistic effects, offering enhanced therapeutic potential. Another critical area for future study is the improvement in the bioavailability of quercetin, which could further enhance its therapeutic benefits. Given the current gaps in our understanding of its mechanisms, the information presented here could guide future research on quercetin to develop new therapeutic strategies for diabetic cardiomyopathy.

## Figures and Tables

**Table 1:** Effects of quercetin on hyperglycemia and associated parameters.

Model	Dose of Quercetin	Findings	Reference
STZ-induced diabetic mice fed high-caloric diet	50 mg/kg/d, i.g., for 4 weeks	↓ blood glucose	[9]
STZ-induced diabetic rats	50 mg/kg, p.o., for 2 months	↔ blood glucose ↔ serum insulin	[35]
STZ-induced diabetic rats	25 mg/kg, i.p., for 30 days	↓ blood glucose	[36]
STZ-nicotinamide-induced diabetic rats	10 mg/kg/d, p.o., for 30 days	↓ blood glucose	[37]
High-cholesterol diet-induced diabetic rats	0.5% w/w diet (5 g/kg diet) for 4 weeks	↓ blood glucose	[38]
Zucker diabetic rats	20 mg/kg/day, in biscuits for 6 weeks	↔ blood glucose	[39]
STZ-induced diabetic rats	10 mg/kg/day, i.p., for 14 days	↔ blood glucose	[40]
I/R-induced heart injury in Zucker diabetic fatty rats (6 and 12 months old)	20 mg/kg/day, in biscuits for 6 weeks	↔ blood glucose	[41]
STZ-induced diabetic rats fed high-caloric diet	50 mg/kg/d, i.g., for 8 weeks	↔ blood glucose ↓ IPITT	[42]
STZ-induced diabetic rats fed high-caloric diet	10 and 50 mg/kg/d, i.g., for 8 weeks	↔ blood glucose ↓ IPITT	[43]
STZ-induced diabetic rats	15 mg/kg typical and nanoemulsion quercetin, i.p., for 8 weeks	↓ blood glucose	[44]
STZ-induced diabetic rats fed high-caloric diet	10 and 50 mg/kg/d, p.o., for 8 weeks	↔ blood glucose	[45]
STZ-induced diabetic rats	100 and 300 mg/kg, p.o., for 12 weeks	↔ blood glucose ↓ fructose	[46]
Fructose-induced insulin resistance rats	50 mg/kg, p.o., for 36 days	↓ blood glucose ↓ blood insulin ↓ HOMA, ↓ OGTT AUC	[47]
STZ-induced diabetic rats	10 and 30 mg/kg, p.o., for 28 days	↓ blood glucose	[48]
Nicotinamide + STZ-induced diabetic rats	10, 25, and 50 mg/kg, p.o., for 28 days	↓ blood glucose ↑ serum insulin	[49]
STZ-induced diabetic rats	10 mg/kg, i.p., for 14 days	↔ blood glucose	[50]
STZ-induced diabetic rats	50 mg/kg, i.g., for 60 days	↓ blood glucose ↔ serum insulin, ↓ HbA1c ↓ serum fructosamine ↓ heart AGEs protein ↓ heart RAGE gene, ↓ heart RAGE protein	[51]
STZ-induced diabetic rats	Water soluble quercetin and liposomal quercetin, 10 mg/kg, i.p., for 14 days	↓ blood glucose ↓ HbA1c	[52]
Nicotinamide + STZ-induced diabetic rats	5, 20, and 80 mg/kg, p.o., for 4 weeks	↓ blood glucose ↑ serum insulin	[53]

AGEs, Advanced glycation end-products; HbA1c, glycated hemoglobin; i.g., intragastric; i.p., intraperitoneal; IPITT, intraperitoneal insulin tolerance test; HOMA, homeostasis model assessment of insulin resistance index; OGTT AUC, oral glucose tolerance test area under the curve; p.o., peroral; RAGE, receptor for advanced glycation end-products; STZ, streptozotocin; ↓, reduced; ↑, increased; ↔, no change.

**Table 2:** Effects of quercetin on lipid metabolism and associated parameters.

Model	Dose of Quercetin	Findings	Reference
STZ-induced diabetic mice fed high-caloric diet	100 mg/kg/d, p.o., for 4 months	↓ plasma TC, ↔ plasma TG	[10]
STZ-nicotinamide-induced diabetic rats	10 mg/kg/d, p.o., for 30 days	↓ Plasma TC, ↓ Plasma LDL ↑ Plasma HDL	[37]
High-cholesterol diet-induced diabetic rats	0.5% w/w diet (5 g/kg diet) for 4 weeks	↓ plasma TC, ↓ plasma TG ↓ plasma LDL, ↓ plasma VLDL ↑ plasma HDL, ↓ heart cholesterol ↓ PPARγ	[38]
Zucker diabetic rats	20 mg/kg/day, in biscuits for 6 weeks	↔ plasma TG, ↔ plasma TC ↔ plasma HDL, ↔ plasma LDL	[39]
I/R-induced heart injury in Zucker diabetic fatty rats (6- and 12-month-old)	20 mg/kg/day, in biscuits for 6 weeks	6-month-old ↔ plasma TG, TC, HDL ↔ plasma LDL 12 months old ↔ plasma TG, TC, HDL ↓ plasma LDL	[41]
STZ-induced diabetic rats	15 mg/kg typical and nanoemulsion quercetin, i.p., for 8 weeks	↓ atherogenic index ↓ cardiac risk ratio ↓ atherogenic coefficient	[44]
STZ-induced diabetic rats	100 and 300 mg/kg, p.o., for 12 weeks	300 mg/kg: ↓ plasma TG, ↓ plasma TC	[46]
Fructose-induced insulin resistance rats	50 mg.kg, p.o., for 36 days (concurrent treatment)	↓ plasma TG, ↓ plasma TC ↑ plasma HDL	[47]
STZ-induced diabetic rats	10 and 30 mg/kg, p.o., for 28 days	↓ plasma TG, ↓ plasma TC	[48]
STZ-induced diabetic rats	Water soluble quercetin and liposomal quercetin, 10 mg/kg, i.p., for 14 days	↓ serum TC, ↓ serum TG ↓ serum LDL, ↑ serum HDL	[68]
Women with type 2 diabetes mellitus (35−55 years old), n = 72	500 mg/day for 10 weeks	↔ plasma TG, ↔ plasma TC ↔ plasma HDL, ↔ plasma LDL	[69]

HDL, High-density lipoprotein; i.p., intraperitoneal; I/R, ischemia/reperfusion; LDL, low-density lipoprotein; p.o., peroral; PPARγ, peroxisome proliferator-activated receptor-γ; STZ, streptozotocin; TC, total cholesterol; TG, triglyceride; VLDL, very low-density lipoprotein; ↓, reduced; ↑, increased; ↔, no change.

**Table 3:** Effects of quercetin on myocardial oxidative stress and inflammation.

Model	Dose of Quercetin	Findings	Reference
H9c2 cells incubated in high glucose	50 μM for 48 h	↓ ROS	[9]
STZ-induced diabetic mice fed high-caloric diet	100 mg/kg/d, p.o., for 4 months	↓ ROS, ↓ NLPR3 protein ↓ IL-1β protein, ↔ IL-18 protein	[10]
STZ-induced diabetic mice fed high-caloric diet	160 mg/kg, p.o., for 6 months	↓ NLRP3 protein ↓ IL-1β protein, ↓ IL-18 protein ↑ SOD protein, ↑ nuc Nrf2 protein ↑ HO-1 protein, ↑ GCLC protein	[11]
H9c2 cells incubated in high glucose	12 µM	↓ NLRP3 protein, ↑ GCLC protein ↓ IL-18 protein, ↓ IL-1β protein ↑ SOD protein, ↓ ROS ↑ nuc Nrf2 protein, ↑ HO-1 protein	[11]
HL-1 cells incubated in high glucose	150 mg/L	↓ ROS, ↓ MDA, ↓ NLRP3 ↑ SOD, ↑ GSH, ↑ TrxR	[18]
STZ-induced diabetic rats	50 mg/kg, p.o., for 2 months	↑ Prx-3 protein, ↔ Prx-5 protein ↑ TrxR2 activity, ↑ Trx-2 protein ↑ Nrf1 protein, ↑ Nrf2 protein ↓ 4-HNE, ↔ GPx activity ↑ SOD activity, ↑ catalase activity	[35]
H9c2 cells exposed to high glucose	1 and 10 μM for 30 min (pretreatment)	↑ Prx-3 protein ↑ nuclei Nrf2 protein	[35]
STZ-induced diabetic rats	25 mg/kg, i.p., for 30 days	↓ inflammatory cells	[36]
High-cholesterol diet-induced diabetic rats	0.5% w/w diet (5 g/kg diet) for 4 weeks	↑ GSH/GSSG content ↑ nuclear Nrf2, ↑ *HO-1* gene ↑ SOD activity, ↑ GPx activity ↑ catalase activity ↓ 8-Isoprostane, ↓ TBARSs	[38]
Zucker diabetic rats	20 mg/kg/day, in biscuits for 6 weeks	↓ SOD, ↔ SOD-1 protein ↑ SOD-2 protein	[39]
STZ-induced diabetic rats	10 mg/kg/day, i.p., for 14 days	↓ SOD, ↓ catalase ↔ GRx, ↔ GPx, ↓ GSH, ↔ GSSG	[40]
STZ-induced diabetic rats fed high-caloric diet	10 and 50 mg/kg/d, i.g., for 8 weeks	↓ ROS, ↓ mt AOPPs, ↓ mt Mn-SOD ↓ mt GPx, ↓ mt GRx ↓ mt NOX, ↓ mt XO	[43]
Fructose-induced insulin resistance rats	50 mg.kg, p.o., for 36 days (concurrent treatment)	↓ LPO, ↑ SOD ↑ catalase, ↑ GPx	[47]
STZ-induced diabetic rats	10 and 30 mg/kg, p.o., for 28 days	↓ lipid peroxides	[48]
Nicotinamide + STZ-induced diabetic rats	10, 25, and 50 mg/kg, p.o., for 28 days	↓ lipid peroxides ↑ catalase activity and protein ↑ SOD activity and protein ↑ GPx activity and protein ↓ NF-κB p65, ↓ IKK-β protein ↓ TNF-α level and protein ↓ IL-1β, ↓ IL-6	[49]
STZ-induced diabetic rats	10 mg/kg, i.p., for 14 days	↔ lipid peroxidation, ↔ SOD, ↑ catalase ↔ GRx, ↔ GPx, ↔ GSH, ↔ GSSG	[50]
STZ-induced diabetic rats	50 mg/kg, i.g., for 60 days	↑ cyt NF-κB protein ↓ nuc NF-κB protein	[51]
STZ-induced diabetic rats	20 and 80 mg/kg, p.o., for 4 weeks	↑ Nrf2 expression	[53]
STZ-induced diabetic rats	Water soluble quercetin and liposomal quercetin, 10 mg/kg, i.p., for 14 days	↓ lipid peroxides, ↓ TBA-active products ↓ diene conjugates ↓ SOD, ↓ catalase, ↑ GSH	[68]
I/R-induced myocardial injury in STZ-induced diabetic rats	5 and 10 mg/kg, i.p., 10 min before reperfusion	Both doses: ↓ MDA, ↑ SOD, ↑ catalase	[72]
I/R-induced myocardial injury in STZ-induced diabetic rats	5 and 10 mg/kg, i.p., 10 min before reperfusion	↓ MPO, ↓ IL-12	[73]

AOPPs, Advanced oxidation protein products; cyt, cytoplasm; GCLC, recombinant glutamate–cysteine ligase; GPx, glutathione peroxidase; GRx, glutathione reductase; HO-1, heme oxygenase 1; IL-1β, Interleukin 1β; i.p., intraperitoneal; I/R, ischemia/reperfusion; LPO, lipid peroxidation; MPO, myeloperoxidase; mt, mitochondria; NLRP3, nucleotide-binding oligomerization domain-like receptor family pyrin domain-containing 3; NOX, NADPH oxidase; Nrf2, nuclear factor erythroid 2-related factor 2; nuc, nucleus; p.o., peroral; Prx-3, peroxiredoxin-3; ROS, reactive oxygen species; SOD, superoxide dismutase; STZ, streptozotocin; TBARSs, thiobarbituric acid reactive substances; Trx-2, thioredoxin-2; TrxR2, thioredoxin reductase 2; XO, xanthine oxidase; ↓, reduced; ↑, increased; ↔, no change.

**Table 4:** Effects of quercetin on cardiac fibrosis.

Model	Dose of Quercetin	Findings	Reference
Zucker diabetic fatty rats (fa/fa)	20 mg/kg/day, p.o., for 6 weeks	↓ LV collagen content ↓ MEF2 protein, ↓ HDAC4 protein ↓ pSer^246^-HDAC4 protein	[8]
STZ-induced diabetic mice fed high-caloric diet	100 mg/kg/d, p.o., for 4 months	↓ *collagen 1* gene ↓ *CTGF* gene	[10]
STZ-induced diabetic mice fed high-caloric diet	160 mg/kg, p.o., for 6 months	↓ Masson-stained area	[11]
Zucker diabetic rats	20 mg/kg/day, in biscuits for 6 weeks	↔ collagen 1 protein, ↔ MMP-2 ↔ 72 kDa MMP-2 protein ↓ 63 kDa MMP-2 protein ↔ MMP-9, ↔ TIMP-2, ↔ MMP-28	[39]
STZ-induced diabetic rats	50 mg/kg, i.g., for 60 days	↔ *TGFβ1* gene, ↓ *CTGF* gene	[51]

CTGF, Connective tissue growth factor; HDAC4, histone deacetylase 4; i.g., intragastric; LV, left ventricle; MEF2, myocyte enhancer factor 2; MMP-2, matrix metalloproteinase-2; p.o., peroral; STZ, streptozotocin; TGFβ1, transforming growth factor-β1; TIMP-2, tissue inhibitor of metalloproteinase-2; ↓, reduced; ↔, no change.

**Table 5:** Effects of quercetin on myocardial apoptosis.

Model	Dose of Quercetin	Findings	Reference
STZ-induced diabetic mice fed high-caloric diet	50 mg/kg/d, i.g., for 4 weeks	↓ TUNEL ↓ Bax protein, ↑ Bcl-2 protein ↓ Bax/Bcl-2 protein, ↓ caspase-3 protein	[9]
H9c2 cells incubated in high glucose	50 μM for 48 h	↓ Bax protein, ↑ Bcl-2 protein ↓ Bax/Bcl-2 protein ↓ caspase-3 protein, ↓ apoptosis rate	[9]
STZ-induced diabetic mice fed high-caloric diet	100 mg/kg/d, p.o., for 4 months	↓ caspase-1 protein	[10]
STZ-induced diabetic mice fed high-caloric diet	160 mg/kg, p.o., for 6 months	↓ caspase-1 protein	[11]
H9c2 cells incubated in high glucose	12 µM	↓ caspase-1 protein	[11]
HL-1 cells incubated in high glucose	150 mg/L	↓ total apoptosis	[18]
STZ-induced diabetic rats	50 mg/kg, p.o., for 2 months	↑ Bcl2/Bax protein, ↓ cyt c protein ↓ TUNEL +ve nuclei ↓ caspase-3 activity, ↓ caspase-9 activity	[35]
STZ-induced diabetic rats	25 mg/kg, i.p., for 30 days	↓ caspase-3	[36]
STZ-induced diabetic rats	10 and 30 mg/kg, p.o., for 28 days	↓ mt cyt c release, ↓ caspase 3, ↓ caspase 9	[48]
Nicotinamide + STZ-induced diabetic rats	10, 25, and 50 mg/kg, p.o., for 28 days	↓ caspase 3 activity and protein ↓ caspase 9 activity, ↑ Bcl-2 protein ↓ Bax protein	[49]

Bcl-2, B-cell lymphoma-2; Bax, Bcl-2-associated X protein; Cyt c, cytochrome c; i.g., intragastric; mt, mitochondria; STZ, streptozotocin; TUNEL, terminal deoxynucleotidyl transferase dUTP nick-end labeling; p.o., peroral; +ve, positive; ↓, reduced; ↑, increased.

**Table 6:** Effects of quercetin on mitochondrial autophagy in the myocardium.

Model	Dose of Quercetin	Findings	Reference
STZ-induced diabetic mice fed high-caloric diet	50 mg/kg/d, i.g., for 4 weeks	↑ LC3II/I protein, ↑ Beclin-1 protein ↓ p62 protein, ↑ p-AMPK, ↓ p-mTOR	[9]
H9c2 cells incubated in high glucose	50 μM for 48 h	↑ LC3II/I protein, ↑ Beclin-1 protein ↓ p62 protein, ↑ GFP-LC3 ↓ MTOR, ↑ p-AMPK, ↓ p-mTOR	[9]

GFP, Green fluorescent protein; i.g., intragastric; LC3, microtubule-associated protein light chain 3; MTOR or mTOR, mechanistic target of rapamycin gene; p-AMPK, phosphorylated adenosine monophosphate-activated protein kinase; p-mTOR, phosphorylated mechanistic target of rapamycin; STZ, streptozotocin; ↓, reduced; ↑, increased.

**Table 7:** Effects of quercetin on cardiac mitochondrial function and energy metabolism.

Model	Dose of Quercetin	Findings	Reference
HL-1 cells incubated in high glucose	150 mg/L	↑ *SIRT5* gene, ↑ *IDH2* gene ↓ IDH2 succinylation, ↑ respiratory complex I ↑ respiratory complex III, ↑ respiratory complex IV ↓ mPTP opening rate, ↑ ATP level ↑ basal respiration, ↑ max respiration ↑ reserve respiration ↓ *Drp1* gene, ↓ *Fis1* gene, ↑ *Mfn1* gene ↑ *Mfn2* gene, ↑ *PGC1α* gene, ↑ *Tfam* gene	[18]
STZ-induced diabetic rats	50 mg/kg, p.o., for 2 months	↓ UCP-3 protein	[35]
High-cholesterol diet-induced diabetic rats	0.5% w/w diet (5 g/kg diet) for 4 weeks	↑ ATP, ↑ *PGC1α* gene, ↓ *UCP-2* gene	[38]
STZ-induced diabetic rats fed high-caloric diet	50 mg/kg/d, i.g., for 8 weeks	↑ Thioredoxin reductase, ↓ AOPPs ↑ aconitase, ↑ succinate dehydrogenase ↓ mitochondrial swelling rate	[42]

AOPPs, Advanced oxidative protein products; ATP, adenosine triphosphate; Drp1, dynamin-related protein 1; Fis1, fission 1; IDH2, NADPH+-dependent isocitrate dehydrogenase 2; i.g., intragastric; Mfn, mitofusin; mPTP, permeability transition pore; PGC1α, peroxisome proliferator-activated receptor gamma coactivator 1-alpha; p.o., peroral; STZ, streptozotocin; SIRT5, sirtuin 5; Tfam; mitochondrial transcription factor A; UCP, uncoupling protein; ↓, reduced; ↑, increased.

**Table 8:** Effects of quercetin on cardiac structure.

Model	Dose of Quercetin	Findings	Reference
Zucker diabetic fatty rats (fa/fa)	20 mg/kg/day, p.o., for 6 weeks	↓ IVSd, ↓ LVPWd, ↓ RWT ↑ LVIDd, ↔ proBNP ↓ GATA4 protein, ↓ SRF protein ↔ total Erk1/2 protein ↔ pErk1/2 protein, ↓ Erk5 protein ↔ PPP1CB, ↔ PPA2C, ↔ pan-Akt	[8]
STZ-induced diabetic mice fed high-caloric diet	50 mg/kg/d, i.g., for 4 weeks	↓ LVEDD, ↓ LVESD ↓ LVW. ↓ LVW/BW	[9]
STZ-induced diabetic mice fed high-caloric diet	100 mg/kg/d, p.o., for 4 months	↓ CK-MB	[10]
STZ-induced diabetic mice fed high-caloric diet	160 mg/kg, p.o., for 6 months	↓ BNP	[11]
STZ-induced diabetic rats	50 mg/kg, p.o., for 2 months	↓ LVSP, ↔ HW/BW, ↓ cTnI protein	[35]
STZ-induced diabetic rats	25 mg/kg, i.p., for 30 days	↓ necrosis, ↓ vacuolization ↓ congestion, ↓ myofibril loss	[36]
High-cholesterol diet-induced diabetic rats	0.5% w/w diet (5 g/kg diet) for 4 weeks	↓ HW/BW, ↔ AWT ↔ FS, ↔ HR, ↔ LVEDC, ↔ LVESC ↔ RVEDC, ↔ RVESC ↔ RV thickness, ↓ LV thickness ↔ septum thickness ↔ RV luminal area, ↑ LV luminal area ↓ RV myocyte density ↓ septal myocyte density ↔ LV myocyte density	[38]
I/R-induced heart injury in Zucker diabetic fatty rats (6 and 12 months old)	20 mg/kg/day, in biscuits for 6 weeks	↔ LV necrosis	[41]
STZ-induced diabetic rats	15 mg/kg typical and nanoemulsion quercetin, i.p., for 8 weeks	↓ troponin I, ↓ CK-MB	[43]
Fructose-induced insulin resistance rats	50 mg.kg, p.o., for 36 days (concurrent treatment)	↓ CK-MB	[47]
Nicotinamide + STZ-induced diabetic rats	10, 25, and 50 mg/kg, p.o., for 28 days	↑ HW/BW, ↓ troponin C ↓ CK-MB, ↓ LDH	[49]
STZ-induced diabetic rats	50 mg/kg, i.g., for 60 days	↓ BNP gene	[51]
STZ-induced diabetic rats	Water soluble quercetin and liposomal quercetin, 10 mg/kg, i.p., for 14 days	↓ HW/BW	[52]
I/R-induced myocardial injury in STZ-induced diabetic rats	5 and 10 mg/kg, i.p., 10 min before perfusion	Both doses: ↓ LV necrosis	[72]
I/R-induced myocardial injury + L-NAME in STZ-induced diabetic rats	5 and 10 mg/kg, i.p., 10 min before perfusion	Both doses: ↓ LV necrosis	[94]

AWT, Aortic wall thickness; BNP, brain natriuretic peptide; cTnI, cardiac troponin I; GATA4, guanine–adenine–thymine–adenine 4; HW/BW, heart weight-to-body weight ratio; i.p., intraperitoneal; I/R, ischemia/reperfusion; IVSd, interventricular septal thickness in end-diastole; LDH, lactate dehydrogenase; L-NAME, Nω-nitro-L-arginine methyl ester; LV, left ventricle; LVEDC, left ventricular end-diastolic cavity; LVEDD, left ventricular end-diastolic diameter; LVESC, left ventricular end-systolic cavity; LVIDd, left ventricular internal diameter in end-diastole; LVPWd, left ventricular posterior wall thickness in end-diastole; LVSP, left ventricular systolic pressure; LVW, left ventricular weight; p.o., peroral; PPA2C, protein phosphatase 2A catalytic subunit; PPP1CB, protein phosphatase 1 catalytic subunit beta; RV, right ventricle; RVEDC, right ventricular end-diastolic cavity; RVESC, right ventricular end-systolic cavity; RWT, relative wall thickness; SRF, serum response factor; STZ, streptozotocin; ↓, reduced; ↑, increased; ↔, no change.

**Table 9:** Effects of quercetin on cardiac function.

Model	Dose of Quercetin	Findings	Reference
Zucker diabetic fatty rats (fa/fa)	20 mg/kg/day, p.o., for 6 weeks	↓ E/A, ↓ FS, ↓ EDV, ↓ ESV	[8]
STZ-induced diabetic mice fed high-caloric diet	50 mg/kg/d, i.g., for 4 weeks	↓ arterial pressure, ↑ EF, ↑ FS ↑ current density, ↓ APD50, ↓ APD90	[9]
STZ-induced diabetic mice fed high-caloric diet	100 mg/kg/d, p.o., for 4 months	↑ EF, ↑ FS, ↔ LVSV, ↔ LVDV	[10]
STZ-induced diabetic rats	50 mg/kg, p.o., for 2 months	↔ HR, ↔ QRS interval ↓ QT interval, ↓ QTc ↓ T peak T end interval ↓ MABP, ↓ LVSP ↑ LV −dp/dt_min_,↑ LV −dp/dt_min_/LVSP ↑ contractility index	[35]
High-cholesterol diet-induced diabetic rats	0.5% w/w diet (5 g/kg diet) for 4 weeks	↓ A wave, ↑ E/A, ↔ EF ↔ FS, ↔ HR, ↔ V_max_ ↔ V_mean_, ↔ IVRT	[38]
Zucker diabetic rats	20 mg/kg/day, in biscuits for 6 weeks	↔ ± dp/dt_max_, ↔ LVDP ↔ RPP, ↔ CF, ↔ HR	[39]
I/R-induced heart injury in Zucker diabetic fatty rats (12 months old)	20 mg/kg/day, in biscuits for 6 weeks	↔ systolic BP (pre-I/R): ↓ LVDP, ↓ +dp/dt_max_, ↓ −(dp/dt)_max_ ↑ HR, ↓ CF (post-I/R): ↓ LVDP, ↔ infarct size	[41]
STZ-induced diabetic rats fed high-caloric diet	10 and 50 mg/kg/d, p.o., for 8 weeks	10 mg/kg: ↑ T-P interval, ↓ T-wave duration 50 mg/kg: ↑ R-R interval, ↓ HR ↑ T-P interval, ↓ T-wave duration	[45]
STZ-induced diabetic rats	100 and 300 mg/kg, p.o., for 12 weeks	↑ E/A, ↑ EF, ↑ FS	[46]
Nicotinamide + STZ-induced diabetic rats	25, and 50 mg/kg, p.o., for 28 days	↑ SBP, ↑ DBP, ↑ HR, ↑ MAP	[49]
STZ-induced diabetic rats	Water soluble quercetin and liposomal quercetin, 10 mg/kg, i.p., for 14 days	↑ HR, ↓ R-R interval ↑ amplitude of R-R interval ↑ VEI, ↑ VRI, ↑ RPAI	[52]
Women with type 2 diabetes mellitus (35−55 years old)	500 mg/day for 10 weeks	↓ SBP, ↔ DBP	[69]

APD50, Action potential at 50% repolarization; APD90, action potential at 90% repolarization; CF, coronary flow; ±dp/dtmax, maximal rates of pressure development/fall; E/A, ratio of peak velocity of early to late filling of mitral inflow; HR, heart rate; i.g., intragastric; i.p., intraperitoneal; I/R, ischemia/reperfusion; IVRT, isovolumetric relaxation time; LVDP, left ventricular developed pressure; LVDV, left ventricular diastolic volume; LVSV, left ventricular systolic volume; MABP, mean arterial blood pressure; p.o., peroral; RPAI, regulatory process adequacy; RPP, rate pressure product; STZ, streptozotocin; VEI, vegetative equilibrium index; VRI, vegetative rhythm indicator; V_max_, maximal velocity; V_mean_, mean velocity; ↓, reduced; ↑, increased; ↔, no change.

**Table 10:** Effects of quercetin on myocardial calcium regulation.

Model	Dose of Quercetin	Findings	Reference
Zucker diabetic fatty rats (fa/fa)	20 mg/kg/day, p.o., for 6 weeks	↓ calcineurin A protein ↓ NFAT3 protein ↔ CaMKII protein ↔ pThr^286^ CaMKII protein	[8]
Isolated hearts from STZ-induced diabetic rats	1 µM in perfusion	↔ TPK Ca^2+^ transient ↓ THALF Ca^2+^ transient ↓ amplitude of Ca^2+^ transient ↓ myofilament sensitivity to Ca^2+^ ↔ SR Ca^2+^ release ↔ resting cell length ↓ TPK shortening ↔ THALF shortening ↓ amplitude of shortening	[100]
STZ-induced diabetic mice	10 and 30 µM perfusion for 30 min in isolated hearts	Both doses: ↑ relaxation time, ↔ contractile force 30 µM: ↓ contraction time	[101]

CaMKII, Calcium/calmodulin-dependent protein kinase II; NFAT3, nuclear factor of activated T cells; SR, sarcoplasmic reticulum; STZ, streptozotocin; THALF, time to half decay; TPK, time to peak; p.o., peroral; ↓, reduced; ↑, increased; ↔, no change.

**Table 11:** Meta-analysis of the effects of quercetin on cardiovascular health in human subjects with metabolic syndrome.

Design	Findings	Reference
17 trials; 896 subjects with metabolic syndrome	Pooled data analysis: ↔ blood lipids, ↔ blood glucose Subgroup analysis in subjects taking quercetin > 8 weeks: ↑ HDL, ↓ TG	[110]
39 trials; 1,501,645 subjects with cardiovascular disease	↓ CHD and CVD risk	[111]
18 trials; 530 subjects (16–1200 mg) with metabolic syndrome	↓ blood lipids, ↓ blood glucose ↓ global risk of CVD	[112]
20 trials; 1164 subjects with metabolic syndrome	↓ blood glucose, ↔ TG, ↔ HDL	[113]
16 trials; 831 subjects with mild metabolic disturbances (quercetin present in buckwheat)	↔ TC, ↔ blood glucose	[114]
8 trials on subjects with metabolic syndrome	↔ VCAM-1, ↔ ICAM-1	[115]

CHD, Coronary heart disease; CVD, cardiovascular disease; HDL, high-density lipoprotein; TC, total cholesterol; TG, triglyceride; VCAM-1, vascular cell adhesion molecule-1; ↓, reduced; ↑, increased; ↔, no change.

## Data Availability

Not applicable.
